# Reproducibility of academic preclinical translational research: lessons from the development of Hedgehog pathway inhibitors to treat cancer

**DOI:** 10.1098/rsob.180098

**Published:** 2018-08-01

**Authors:** Tom Curran

**Affiliations:** Children's Research Institute, Children's Mercy Kansas City, 2401 Gillham Road, Kansas City, MI 64108, USA

**Keywords:** translational research, cancer, Hedgehog pathway, reproducibility, unconscious bias

## Abstract

Academic translational research is growing at a great pace at a time in which questions have been raised about the reproducibility of preclinical findings. The development of Hedgehog (HH) pathway inhibitors for the treatment of cancer over the past two decades offers a case study for understanding the root causes of failure to predict clinical outcomes arising from academic preclinical translational research. Although such inhibitors were once hoped to be efficacious in up to 25% of human cancer, clinical studies showed responses only in basal cell carcinoma and the HH subtype of medulloblastoma. Close examination of the published studies reveals limitations in the models used, lack of quantitative standards, utilization of high drug concentrations associated with non-specific toxicities and improper use of cell line and mouse models. In part, these issues arise from scientific complexity, for example, the failure of tumour cell lines to maintain HH pathway activity *in vitro*, but a greater contributing factor appears to be the influence of unconscious bias. There was a strong expectation that HH pathway inhibitors would make a profound impact on human cancer and experiments were designed with this assumption in mind.

## 1. Introduction

Over the past two decades, much of the burden for preclinical research, and even early clinical trials, has shifted from industrial to academic laboratories, as companies conserve their resources. There is now a great demand by industry for academic laboratories to de-risk projects, by providing strong indications of efficacy, before partnerships are formed. However, increasingly, questions are being raised about the lack of reproducibility of academic translational research findings in industrial laboratories [1]. This has led to calls for greater education in statistical methodology, strategies to verify reproducibility and the establishment of publication watchdog websites, such as *PubPeer* and *Retraction Watch*, dedicated to pointing out deficiencies in the published literature. However, each of these initiatives fails to deal with the root problem. Rather than provide a theoretical discourse of the issues, I will review key steps involved in the development of small molecule inhibitors of the Hedgehog (HH) pathway for the treatment of cancer, to illustrate pragmatic lessons in preclinical translational research. Although the development of HH pathway inhibitors for the treatment of cancer was a successful translational journey, leading to drug approvals, there were many instructive failures along the way. These missteps point to flawed reasoning, improper use of models, lack of quantitative scientific approaches and self-delusion, each of which stem from pervasive unconscious bias in academic translational research. To illuminate these issues, it is necessary to dig into the details of the science to understand, and hopefully avoid, similar problems in the future.

## 2. The difference between translational and basic research

There is a fundamental difference between translational research and basic research. In basic research, investigators may favour a particular hypothesis, but they strive to maintain disinterest while testing their ideas through critical experimentation that advances knowledge regardless of the outcome. In translational research, the goal is to generate positive results that support the development of a product, or intervention strategy, to improve patient outcomes. In experimental therapeutics, the stakes can be very high during the preclinical research stage, carried out to support the decision to advance a product into the clinic. Similarly, in phase I/II clinical trials, there can be enormous negative consequences if a product progresses only to fail at the later phase III stage, or beyond. In industry, great efforts are made to challenge projects as early as possible in the pipeline because of the financial and lost opportunity costs of late-stage failure. Indeed, company scientists are often rewarded for uncovering scientific flaws, off-target effects or unexpected toxicities that result in shutting down a project before it consumes inordinate resources. However, this incentive does not exist in academia and therein lies the problem.

The currency of academic research is publications and grants. Notoriously, it is very challenging to publish negative data and grants are not awarded, or renewed, when a project fails to show promising results. This means that embarking on a multi-year translational study is a high-risk endeavour for the entire research team. Students need publications to graduate, fellows require high-impact studies to open the door to an independent career and laboratory heads must constantly generate grant revenue to survive. In this climate, it is almost impossible to maintain disinterest. Even if this perspective can be maintained at the conscious level, wishful thinking creeps in because of unconscious bias.

## 3. Target identification

The first step in any drug development project is to identify a target. In the case of the HH pathway, the target was uncovered as a result of the gene mapping studies, demonstrating that basal cell nevus carcinoma syndrome (BCNS), also known as Gorlin syndrome, is a consequence of mutations in Patched-1 (PTCH1), the receptor for HH ligands [2,3]. In addition to developmental defects, BCNS is associated with a high frequency of basal cell carcinoma (BCC) and an elevated incidence of the paediatric brain tumour medulloblastoma (MB) [4]. Germline loss of one copy of *PTCH1* is complemented by somatic mutation, or silencing, of the remaining allele in tumour cells, revealing that *PTCH1* acts like a classic tumour suppressor gene in BCC and MB. These initial reports were followed quickly by studies identifying *PTCH1* mutations in sporadic BCC and MB [5]. It is now clear that other mutations in the pathway, including gain-of-function mutations in Smoothened (*SMO*) and loss of Suppressor-of-Fused (*SUFU*), also result in BCC and MB [6]. In all cases, these mutations result in high levels of HH pathway activity, independent of ligands, leading to elevated expression of downstream transcriptional target genes, including *GLI1* and *GLI2*.

The work progressed rapidly, largely due to the prior decades of outstanding basic research, initiated by pioneering work in *Drosophila* genetics [7] that was recognized by the award of a Nobel Prize to Christiane Nusslein-Voldhard, Eric Wieschaus and Edward Lewis. This work was extended by high-quality developmental biology studies that elucidated the critical role of the HH pathway in a broad range of developing tissues [8]. It is important to stress that quality translational research is built on quality basic research, and we must continue to interpret translational research findings in the context of detailed knowledge of the biology of the target. This was particularly important in the development of SMO inhibitors, as fears of developmental bone toxicities, because of the well-known role of the HH pathway in the bone growth plate [9], were borne out in the clinic [10,11]. This resulted in a Federal Drug Administration restriction on the use of SMO inhibitors in young children prior to completion of bone growth that, unfortunately, was only put in place after bone malformations, first described in young mice [12,13], were recapitulated in children.

**Figure 1.**
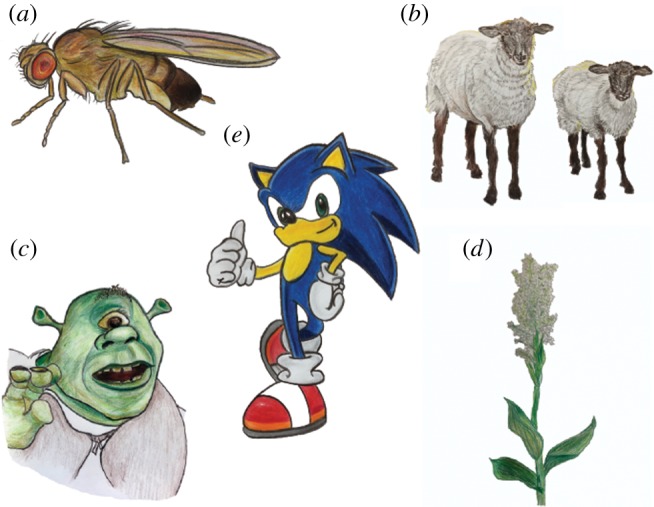
Flies, sheep, corn lilies, cyclops and Sonic the Hedgehog point the way to novel cancer therapeutics. Early genetic studies in *Drosophila* (*a*) described the role of the HH pathway in development and later this was extended to mammals. Mutations in the HH gene cause holoprosencephaly (cyclops-like phenotype) in mice, sheep (*b*), humans and mythical beasts (*c*). The same phenotype arises in lambs born to ewes that ingest the corn lily plant (*V. californicum*) (*d*) in the first trimester. This was the clue that ultimately led to the identification of small molecule inhibitors of the HH pathway. The mammalian HH gene family includes SHH, a name inspired by the eponymous video game character designed by Sega Inc. (*e*). Illustrations by T. Curran.

The identification of *PTCH1* loss as a therapeutic target presented a conundrum—because it is deleted from tumour cells, how does one target an absence? The solution was revealed by a remarkable series of observations that, when tied together, read like clues from a detective novel (figure 1). The first clue was the observation of holoprosencephaly in lambs, caused by ingestion of corn lilies (*Veratrum californicum*) by pregnant ewes [14]. Ultimately, the teratogens were identified as plant steroidal alkaloids [15] and one of these, termed cyclopamine because of its ability to induce holoprosencephaly (the cyclops-like phenotype known from ancient times), was also shown to induce limb malformations [16]. Similar defects occur as a consequence of genetic mutations in *Sonic Hedgehog* (*SHH*) and other components of the HH pathway in a range of species, including humans [17]. Cyclopamine was then shown to function as an inhibitor of the HH pathway [18,19]. Thus, the naturally occurring teratogen, cyclopamine, induces a phenocopy of certain HH pathway mutations. The key finding that tied the threads of the story together was the observation from the Beachy Laboratory that cyclopamine functions by direct binding to SMO to shut down HH pathway activity [20].

**Figure 2.**
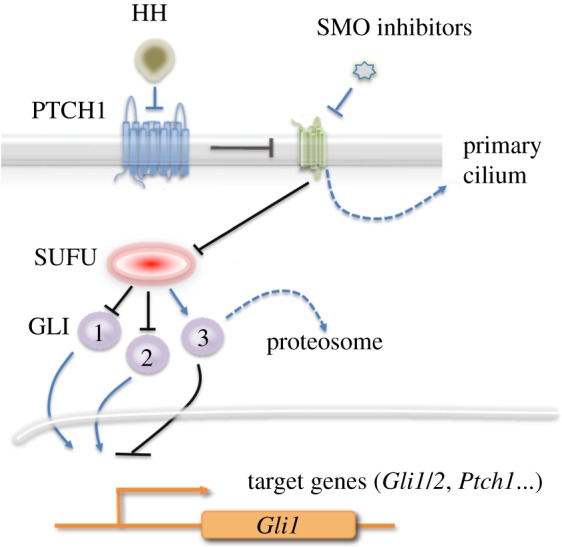
The HH signalling pathway. HH ligands bind to the membrane-associated protein PTCH1 and inhibit its function. PTCH1 inhibits SMO by preventing it from translocating to the primary cilium. SMO inhibits SUFU which, in turn, inhibits the activation and translocation of GLI1 and GLI2 to the nucleus. SUFU also activates GLI3, which is processed by proteolytic cleavage to become a transcriptional repressor. GLI1 and GLI2 activate transcription of several target genes, including themselves and *PTCH1*. The loss of PTCH leads to constitutive activation of the pathway. Small molecule inhibitors bind and inhibit SMO.

The complexity of the HH pathway continues to evolve to the present day. Briefly, HH ligands (Sonic, Indian and Desert) bind and inhibit PTCH1 (figure 2). PTCH1 is a negative regulator of SMO. Thus, in tumours cells lacking PTCH1, SMO is constitutively active and HH pathway activity remains elevated. Cyclopamine, and similarly acting compounds, binds to SMO and blocks its function, thereby shutting off HH pathway activity. With the discovery that cyclopamine is an inhibitor of SMO, the focus of many investigators in the field transitioned from a basic research perspective to preclinical translational research. These groups shared the common goal of developing proof-of-concept data, using cell culture and animal models, to support the use of HH pathway inhibitors as anticancer agents in clinical trials in humans. However, although this sounded simple, challenges were encountered immediately that led to overinterpretation of results and unrealistic expectations.

## 4. The use of cancer models

Traditionally, the first step in testing potential anticancer agents is to determine whether they can inhibit the growth of cultured tumour cells. The issue that confronted investigators studying SMO inhibitors was that there were no tumour cell lines available in which activating HH pathway mutations had been documented and elevated HH pathway activity had been demonstrated. Following initial studies in cell culture, the next step would normally be to test the drugs in xenograft models of human tumours, but there were no models in which the status of the HH pathway had been validated. An alternative approach was provided by the *Ptch1^+/−^* mouse strain generated by the Scott Laboratory [21]. These mice develop tumours resembling the desmoplastic subtype of human MB and they harboured an activated HH pathway. The low frequency and sporadic appearance of the tumours were addressed by crossing the mice into a *p53^−/−^* background to generate a strain, *Ptch1^+/−^p53^−/−^* mice, that exhibited a 100% incidence of MB within two weeks of age [22]. The mice were also used to generate a model for BCC by exposing their skin to ultraviolet or ionizing radiation [23].

The first published report, investigating the efficacy of the SMO inhibitor cyclopamine as an anticancer agent, used cultured tumour cells from mice and humans as well as allograft tumours established from mouse tumour cell lines [24]. However, it was shown subsequently that the HH pathway activity is rapidly suppressed when MB cells from *Ptch1^+/−^* mice are cultured *in vitro* [25]. Recently, this was revealed to be a consequence of the loss of tumour-associated astrocytes which maintain HH pathway activity in tumour cells by secreting SHH [26]. Allograft tumours, derived from cultured mouse MB cells, do not harbour an active HH pathway and they fail to respond to SMO inhibitors [25]. So, how was it possible to obtain supportive efficacy data for cyclopamine if the target was not active in the models used?

The well-known problem with cyclopamine is that the concentration of drug required to block the HH pathway is close to the concentration that induces cell death independently of the HH pathway [27]. Culturing mouse and human MB cells in the presence of 3–5 µM cyclopamine (or in the case of the more potent variant KAAD-cyclopamine, 1 µM) for 48–72 h reduced the growth of tumour cells [24]. However, this concentration of cyclopamine is toxic for many cell types. In fact, it has now been demonstrated that cyclopamine promotes apoptosis in the human MB cell line DAOY by inducing expression of neutral sphingomyelin phosphodiesterase 3, which increases ceramide production and induces cell death, independently of the HH pathway [28]. Thus, studies using cultured mouse MB tumour cell lines are hampered by the fact that cyclopamine has strong, off-target toxic effects and that the HH pathway is no longer active in these cells. Initial reports documenting the effects of cyclopamine on embryonic development and inhibition of the HH pathway used drug concentrations as low as 120–130 nM to achieve specific biological effects [18,19]. By contrast, the majority of tumour cell line studies used 5–10 µM, and sometimes up to 20–30 µM cyclopamine, to inhibit growth [29]. This means that most studies were carried out under conditions in which cyclopamine promotes ceramide-induced cell death independently of SMO.

Initial attempts to generate mouse MB tumour cell lines that retain HH pathway activity *in vitro* failed [25]. Although the tumour cells grew readily *in vitro*, they no longer exhibited the HH pathway gene expression signature. Some cell lines did express GLI1, which increased when they were propagated as allografts; however, this turned out to be a consequence of *Gli1* gene amplification but not SMO signalling [25]. Subsequent efforts claimed greater success, but the lines were established at a low frequency (20%) and they exhibited only partial sensitivity to high doses of the SMO inhibitor, LDE225 [30]. Recent studies have revealed that mouse MB tumour cells require the presence of tumour-associated astrocytes to maintain an active HH pathway in culture [26]. These cells are lost when tumour tissue is placed into cell culture and this is why the HH pathway is suppressed *in vitro*.

The other critical experiment in the initial report on the use of cyclopamine as an anticancer agent was the treatment of mice carrying allograft tumours derived from mouse MB cells [24]. Cyclopamine was shown to cause tumour regression in this model. However, because these allografts were derived from mouse MB cells propagated in culture, they should not have harboured an active HH pathway [25]. The method chosen for drug delivery in this study was subcutaneous inoculation of 0.1 ml of cyclopamine suspended in a 4 : 1 mixture of triolein/ethanol [24]. Others reported this approach to cause lesions at the site of injection, forcing premature termination of experiments [31,32]. One concern is that, as the lesions spread due to daily treatment, this may ultimately have led to inoculation of the drug near or even directly into the tumour mass, potentially inhibiting tumour cell growth, not because of suppression of the HH pathway, but as a result of the off-target toxic effects of the high drug concentrations. In a number of cases, alcohol was present in the carrier at a level of 20%, which causes necrosis at the site of inoculation into the tumours [33]. Recognizing this problem, alternative carriers lacking ethanol were developed for cyclopamine. However, the practice of injecting the drug subcutaneously near, or directly into, the tumour was widely adopted [34–36]. A common strategy for xenograft treatment was described in the following way: ‘soon as the tumour was palpable, cyclodextrin-conjugated cyclopamine or cyclodextrin carrier alone (Sigma) at 10 mg kg^−1^ was injected in the immediate vicinity, or intratumorally when possible, twice daily’ ([36], supplemental data p. S6). This process continues to be used today, even though concerns about the procedure were pointed out, including the fact that the drug concentration at the site of injection is extremely high and above the level that induces off-target toxicity [37]. In addition, because the tumour is being treated before it becomes established, the assay is really measuring inhibition of tumour establishment rather than inhibition of tumour growth. Finally, the physical and hydrostatic pressure damage resulting from twice daily inoculations into small tumour volumes may by itself be enough to prevent growth. This approach led to an overestimation of the range of tumour types that appear to respond to SMO inhibitors. The factors discussed above, including the off-target toxicity of cyclopamine, the use of excessively high concentrations of drugs, the lack of common gene expression markers to define HH pathway activation and the direct injection of cyclopamine into tumours, continue to affect the field today [38].

In general, the use of a systemic dosing route is recommended when testing anticancer agents in animal models, for example, by oral gavage or intraperitoneal inoculation. When the oral gavage route was tested for cyclopamine, it was not possible to reach a dose that completely suppressed HH pathway activity, in mice carrying a *Gli*-luciferase reporter transgene, due to toxicity [13]. It is also important to treat established tumours, usually greater than 150 mm^3^, to obtain a reliable measure of tumour regression. Treating transplanted tumour cells before the tumour has been established does not provide reliable data on tumour growth. Rather, it provides information on whether the treatment can prevent engraftment in the host. Although we now know that SMO inhibitors are efficacious in treating a subset of human BCC and MB, it is important to re-examine the initial reports carefully, as these studies established methodological practices that were adopted by the field and are currently employed today.

## 5. Transitioning preclinical research into clinical trials

Several compounds from a range of structural classes, with a much better therapeutic index than cyclopamine, were generated in a high-throughput small molecule screen conducted by Curis Inc. [39]. These compounds inhibited HH pathway activity at nanomolar levels, and they demonstrated efficacy in the *ex vivo* skin punch mouse BCC model from *Ptch1*^+/−^ mice described above [40]. However, it was their ability to eliminate even large spontaneous mouse MB in *Ptch1^+/−^p53^−/−^* that attracted great interest in their potential anticancer agents [41]. In this case, the tested compounds were delivered by oral gavage and shown to cross the blood–brain barrier to block HH pathway activity in brain tumour tissues. Two weeks of treatment, twice daily, with 100 mg kg^−1^ of one of the compounds, termed HhAntag, completely eliminated large MB tumours [41]. Subsequently, it was found that MB, from *Ptch1^+/−^* and *Ptch1^+/−^p53^−/−^* mice, grafted onto the flank of immunosuppressed mice, retained this high level of sensitivity to SMO inhibitors, so that even large tumour masses could be eradicated in less than 5 days of treatment [25]. Because of ease of use, this direct allograft system became the model of choice for many preclinical studies, including those used to support the launch of successful clinical trials and subsequent approvals of vismodegib (Genentech Inc.) and sonedigib (Novartis Inc.) by the Federal Drug Administration. Importantly, no human xenograft studies exhibited this level of response and there remains no validated human xenograft model for HH pathway tumours even now. A large number of companies conducted their own successful small molecule screens, as SMO turned out to be a highly druggable target, leading to the testing of 10 different SMO inhibitors in 86 clinical trials listed on *clinicaltrials.gov*.

In contrast with the use of genetically engineered mouse (GEM) models to develop SMO inhibitors for the treatment of BCC and MB, numerous groups employed human tumour cell lines and xenograft models in preclinical studies to extend the potential application of SMO inhibitors to a broad range of human cancers. Most of the initial studies used cyclopamine as the SMO inhibitor, but, more recently, a broader range of SMO inhibitors have also been employed. These studies followed a familiar path, usually starting by testing the inhibitors on a collection of tumour cell lines to inhibit cell proliferation and induce apoptosis before transitioning into xenograft models. Although none of these other tumours harboured mutations in the HH pathway, evidence of expression of HH pathway genes was interpreted as an indication that the pathway was activated. Invariably, these studies reported evidence of preclinical efficacy, thus paving the way for the 86 clinical trials mentioned above. Several hundred such studies have been published and the following are representative examples of tumour-specific analyses: small cell lung cancer [42], pancreatic cancer [43], colorectal cancer [29], prostate cancer [44], breast cancer [45], hepatocellular carcinoma [46], ovarian carcinoma [47] and glioma [36]. Each of these studies used cyclopamine, at various concentrations up to 20 µM, to induce cell death in tumour cell cultures. Based on these results and others, it was claimed that the HH pathway contributes to approximately 25% of human cancer deaths [48]. Therefore, it was a great disappointment to learn that, despite these positive preclinical studies, clinical responses were reported in only BCC and MB [11,49–53]. Several studies documented the lack of efficacy of SMO inhibitors, alone or in combination with chemotherapy, in a range of tumours [54–58], even though in some cases, a reduction in GLI1 expression levels was observed in tumour tissues. Many other negative results have yet to be reported. The simple explanation for the widespread failure of SMO inhibitors in clinical trials is that the preclinical data used to support the transition into the clinic represented false-positive results.

## 6. Why preclinical studies of SMO inhibitors failed to predict responses in the clinic

Unlike BCC and MB, no activating mutations in HH pathway genes have been reported in the other tumours proposed to be treated with SMO inhibitors [31–38]. Evidence of an HH pathway gene expression signature was used to determine that the pathway was active in these tumours. However, there was no established standard for the level of gene expression required, no definition of which genes should be used to represent the authentic HH pathway signature, and no agreed upon methodology for documenting HH pathway gene expression. This led to each investigator defining their own standards, ultimately leading to confusion over the definition of ‘activated HH pathway’ and how this should be measured. In MB, tumour subsets were identified initially by supervised hierarchical clustering analysis using probes specific for genes whose expression increased in the presence of HH ligands [59]. This approach readily identified the HH-MB subtype and a similar strategy applied to the WNT pathway distinguished tumours with β-catenin mutations [59]. Subsequently, these groups were refined using non-supervised clustering approaches and the genes in the signature were not necessarily transcriptional targets of HH pathway signalling [60,61]. However, for the preclinical studies of SMO inhibitors, investigators generally relied on a select number of HH pathway target genes. In many cases, the level of *GLI1* expression was employed as a quantitative measure of HH pathway activity. However, this can be misleading as *GLI1* can be regulated independently of the HH pathway [62], and it can exhibit an increased copy number in tumour DNA which influences its expression level independently of the HH pathway. In fact, the name *GLI* was coined based on its discovery as an amplified gene in glioma [63].

Although *GLI1* is a transcriptional target gene in the HH pathway, it is not an essential gene for development [64] and MB can arise in the absence of *Gli1* in *Ptch1*^+/−^ mice, albeit at a reduced level [65]. These tumours express high levels of *Gli2* which has overlapping functions with *Gli1*. Thus, the presence and level of *GLI1* expression may not reflect the level of HH pathway activity in tumours. In C3H10T1/2 cells, *GLI1* and *GLI1* reporter constructs are very sensitive indicators of HH pathway activity over a broad dynamic range [39]. SMO inhibitors are capable of essentially eliminating *GLI1* expression and blocking luciferase activity driven by *GLI1-*binding sites over several orders of magnitude [39]. Therefore, observations of only a modest 50% drop in the level of *GLI1* RNA in tumour cells treated with high doses of cyclopamine do not support the argument that the HH pathway is driving both *GLI1* expression and the growth of such cells [66,67]. Instead, these results indicate that cyclopamine may be inhibiting cell growth independently of its effect on SMO and HH pathway activity. Nevertheless, it is important to point out that GLI1 is a bone fide target in some tumours regardless of its role in the HH pathway. Toftgard and co-workers [68] developed a GLI1 inhibitor, GANT61, that is effective at inhibiting tumour cell growth *in vitro* and *in vivo*. In addition, arsenic trioxide (ATO), an approved FDA drug, was shown to bind and inhibit GLI1, causing reduced tumour cell growth *in vitro* and *in vivo* [69,70]. GLI1 inhibitors, like GANT61, may have a much broader indication than SMO inhibitors because GLI1 is expressed in many tumour cells that do not have an active HH pathway. In the latter case, these tumours would not be expected to respond to SMO inhibitors. Similarly, tumours with an activating mutation in the HH pathway downstream of SMO, such as *SUFU* loss, or tumours that develop resistance to SMO inhibitors, may be responsive to GANT61 or similar inhibitors that target GLI1 [71–74]. As GLI1 is neither necessary, nor sufficient, for HH pathway activity, it cannot be relied upon, by itself, to provide a biomarker for the pathway. The lack of a defined, quantitative standard biomarker of the activated HH pathway, coupled with the broader role of GLI1 in tumours, means that the number and diversity of HH pathway tumours has been vastly overestimated.

## 7. Paracrine and autocrine Hedgehog pathway activity

The absence of HH pathway mutations in tumours proposed to have an activated HH pathway led to the suggestion that autocrine signalling by HH ligands was responsible for driving HH pathway activity and tumour growth [43,44,75]. Evidence supporting the overlapping expression of HH pathway genes, including ligands, in tumour cells relied on immunohistochemistry performed using commercially available antibodies that are notoriously difficult to rely on due to lack of specificity. Analysis of RNA from prostate tumours revealed the expression of the HH pathway genes *SHH*, *PTCH1* and *GLI1* at elevated levels compared to surrounding normal tissues in the broad range of 1.5–300-fold [44]. However, there was no correlation among the expression levels of each of these genes, implying that there was no coordination between the level of ligand and that of two different HH pathway transcriptional target genes. There was also significant variation among cell line responses to a high concentration, 10 µM, of cyclopamine [43,44]. Other groups who investigated the same or similar prostate cancer cell lines could not confirm these results, which led them to conclude that there was no evidence of autocrine signalling, although there was evidence that HH ligands secreted by tumour cells were acting on the tumour stroma [76,77].

The controversy was addressed in an exhaustive analysis carried out by de Sauvage and co-workers [78] at Genentech Inc., using a more potent, and less toxic, SMO inhibitor termed HhAntag, in addition to cyclopamine, to investigate paracrine and autocrine HH pathway signalling in tumour cells. This was a remarkable study for a number of reasons. To set the scene, at that moment in time, the role of the HH pathway in cancer was subject to lively discussions at many scientific meetings. In fact, this author participated in an organized debate, with one of the proponents of the paracrine signalling model, at the American Association for Cancer Research annual meeting in 2007. The stakes were relatively high as several major pharmaceutical companies and numerous biotech companies had active programmes designed to develop small molecule inhibitors of SMO. The investigators at Genentech were in an unusual position. As the first major company to develop an HH pathway programme, in collaboration with Curis Inc., it would have been in their interest to find evidence supporting the optimistic view that SMO inhibitors would be efficacious in 25% of human cancer. However, they found no evidence of autocrine signalling in any tumour cell line studied [78]. They did confirm that high concentrations of cyclopamine or HhAntag could inhibit cell growth, but this did not correlate with HH pathway activity and, in the case of HhAntag, they needed to use approximately 400 times higher concentrations than those required to block HH pathway activity. This analysis employed 122 tumour cell lines, many of which were the same lines used by other investigators to reach the opposite conclusion. The authors concluded that the previously reported effects of SMO inhibitors on growth and HH pathway activity in tumour cells were a consequence of off-target effects resulting from the use of the inhibitors at high, non-physiological concentrations.

One cannot help wondering whether this resounding failure to reproduce academic preclinical translational studies, and the fact that 0/122 human cancer cell lines supported a role for the HH pathway in tumour cell growth may have caused corporate leadership some pause before continuing on the path to clinical development.

In addition to studying tumour cell lines, de Sauvage and co-workers [78] also investigated a large collection of xenograft models. They noted that some of the cell lines, and certain human xenografts, did express HH ligands and they demonstrated that the ligands secreted by tumour cells were capable of stimulating HH pathway activity in stromal cells. They went on to show that SMO inhibitors were capable of retarding the growth of some xenograft models by inhibiting HH pathway activity in the stromal environment. However, although this effect was statistically significant, it was relatively modest, and no tumour regression was observed. This low level of growth retardation in xenograft models rarely, if ever, predicts responses in the clinic. The authors remained cautious in their interpretation while acknowledging that the exact mechanism whereby increased HH pathway activity in stromal cells supports tumour growth remained to be determined, the observation raised the potential for the use of SMO inhibitors to target the tumour microenvironment.

The proposed effect of paracrine HH signalling on tumour stroma was also investigated in a GEM model of pancreatic ductal adenocarcinoma [79]. This model was generated by conditional expression of mutant *K-ras* and *p53* genes in pancreatic progenitor cells [80]. The authors hypothesized that HH signalling from tumour cells may support the maintenance of the stromal compartment and that disruption of HH signalling might facilitate the delivery of chemotherapeutic agents. They reported that the combined treatment of mice with gemcitabine and the SMO inhibitor IPI-926 [81] resulted in increased survival from 11 to 25 days [79]. While this result was statistically significant, it represents a relatively modest effect, as all the treated mice died within a few days. Furthermore, the effect was even less compelling when comparing the survival of mice treated with gemcitabine alone with those treated with the drug combination. In addition, not all tumours exhibited a transient decrease in size during the course of treatment. This may be a case of wishful thinking—that such a modest delay in tumour growth would translate into a clinical response in patients. A phase II clinical trial sponsored by Infinity Pharmaceuticals (IPI-926-03), in which patients received either the combination of gemcitabine plus IPI-926 or placebo, was halted early due to poor outcomes. A similar trial, using the combination of gemcitabine and the SMO inhibitor vismodegib, also failed to show improved survival [82]. In a follow-up study of the mouse model, deletion of *Shh* from pancreatic epithelial cells resulted in earlier tumour growth and decreased survival of mice [83]. This study also failed to reproduce prior results obtained by treating tumours with IPI-926 plus gemcitabine. In fact, the investigators found that IPI-926 treatment caused more aggressive tumour growth [83]. These findings indicate that, in this tumour model, SHH is not just dispensable for tumorigenesis, but it actually constrains tumour progression.

## 8. The non-canonical Hedgehog pathway

*Canon*, meaning rule or accepted principle, is derived from the ancient Greek *kanon*—a measuring rod or standard. The standard, or canonical, HH pathway in mammalian cells refers to the genetically and biochemically defined signalling process involving HH ligands, PTCH1, SMO, SUFU and GLI proteins. There are, of course, many other proteins involved in HH signalling, but these are mostly believed to modulate the core components listed above. The lack of correlation between the effects of SMO inhibitors on tumour cell growth and the levels of HH pathway target genes led some investigators to propose that ‘non-canonical’ HH pathway signalling, potentially involving cross-talk with the RAS and TGFβ pathways [84], androgen receptor signalling [85], the mTOR pathway [86] and the WNT pathway [87] among others, contributed to tumour progression. Given the range of contributing factors involved, it has become challenging to distinguish among several possible non-canonical HH pathways. While it is clear that HH signalling influences, and is influenced by, numerous other signalling pathways, it is not easy to determine if these truly constitute non-canonical pathways. The studies referenced above suffer from some of the shortcomings already discussed, including the point that the presence of GLI1 by itself does not imply an activated HH pathway. Therefore, the fact that GLI1 inhibitors like GANT61 may inhibit tumour cell growth, whereas SMO inhibitors have no effect, cannot be used as evidence for a non-canonical HH pathway [68,84]. Instead, these studies indicate that expression of GLI1 does not always require SMO and that GLI1 has intrinsic growth and oncogenic properties, independently of the canonical HH pathway. The other common feature among non-canonical HH pathway studies is the use of SMO inhibitors at high concentrations where they inhibit cell growth through off-target effects.

Recently, signalling by exogenous SHH ligand was shown to occur in MB cells lacking PTCH1 [26]. This effect still required SMO and it was blocked by SMO inhibitors [26]. The source of SHH *in vivo* was shown to be tumour-associated astrocytes. In contrast with the regular HH pathway, in this case, SHH induced expression of *nestin* in mouse MB cells (figure 4). This effect, which could be blocked by SMO inhibitors, resulted in sequestration and inhibition of GLI3, thus abrogating its inhibitory effect on the HH pathway. While the SHH receptor in cells lacking PTCH1 has not yet been identified, PTCH2 has been shown to modulate tumorigenesis in *Ptch1*^+/−^ mice [88]. In other cells, in the absence of PTCH1, PTCH2 mediates the response to SHH [89]. The induction of *nestin* expression by SHH in *Ptch1*^−/−^ cells appears to be independent of GLI1 as it was not promoted by exogenous overexpression of GLI1 [26]. The induction of *nestin* in MB cells by SHH secreted from astrocytes clearly involves a paracrine mechanism. Thus, in the case of mouse MB, tumour progression is associated with a gradual acquisition of *nestin* expression that abrogates a negative constraint on HH pathway activity. It seems that SMO inhibitors may be particularly effective in this mouse MB model as they simultaneously block the intrinsic activity of the HH pathway resulting from *Ptch1* loss as well as the extrinsic effect of SHH secreted by astrocytes. As the induction of *nestin* by SHH signals through SMO, it may not be accurate to refer to this process as a non-canonical pathway. However, its lack of dependence on PTCH1 and GLI1 make it different from the canonical pathway; therefore, it has been referred to as a paradoxical HH pathway [26]. Whatever the name, it appears to play a significant role in the growth of mouse MB and, potentially, human MB.

## 9. The curious case of rhabdomyosarcoma

Rhabdomyosacroma (RMS) is a collection of soft tissue sarcomas, derived from skeletal muscle, that primarily occur in children. They can be very challenging to treat, depending on the subtype [90]. The loss of *PTCH1* in Gorlin syndrome is associated with a range of rare tumours in addition to BCC and MB, including fetal rhabdomyoma [91]. In mice, heterozygous loss of *Ptch1* in CD1 mice is associated with a 9% incidence of tumours resembling the embryonic subtype of RMS (ERMS) [92]. The incidence of these tumours is influenced by genetic modifiers, as they are only rarely encountered on a C57Bl/6 background. Parenthetically, Balmain and co-workers [93] demonstrated that a polymorphic variant in *Ptch1*, present in FVB/N mice but absent in C57BL/6 mice, functions as a genetic modifier to promote RAS-induced squamous cell carcinoma. ERMS tumours from *Ptch1^+/−^* mice display high levels of some HH pathway target genes, including *Gli1*, *Ptch1* and *Igf2* [94]. Several groups have performed detailed preclinical studies on RMS in an effort to determine whether they are good candidates for treatment with HH pathway inhibitors; however, this work has proved challenging and several questions remain [31,92,95–98]. The emerging consensus seems to be that, while such tumours may be targeted by agents that inhibit GLI1, such as GANT61, they are not sensitive to treatment with physiological levels of SMO inhibitors [97].

ERMS in humans has not been linked to mutations in the HH pathway and while there is frequent loss of heterozygosity of chromosome 11p15, the chromosomal translocations indicative of alveolar RMS are not present [99]. Often, HH pathway activity is reported to be elevated in ERMS based primarily on the detection of high levels of *GLI1* and *PTCH1* RNA expression [99,100]. However, this is not the standard gene expression profile that defines an activated HH pathway. The complication is that PTCH1 is a negative regulator of the HH pathway and, as a transcriptional target of the pathway, it participates in a negative feedback loop [101]. This leads to the contradictory observations that high levels of normal *PTCH1* RNA indicate an activated HH pathway but, conversely, the presence of high levels of PTCH1 protein implies that the pathway is inhibited. In BCC, coincidental high expression of *PTCH1* and *GLI1* is only seen in tumours expressing a mutated *PTCH1* allele that is ineffective at suppressing the HH pathway [102]. This is not the case in RMS as *PTCH1* mutations are not present in these tumours [103]. In MB with an activated HH pathway, high *GLI1* expression is associated with low levels of expression of the normal *PTCH1* allele [104]. Thus, the presence of elevated levels of both *PTCH1* and *GLI1* may not imply that the HH pathway is active in ERMS. Hahn and co-workers [97] reported that a series of four SMO inhibitors were ineffective at inhibiting *GLI1* expression in RMS cells and, in some cases, treatment even resulted in elevated expression levels. The change in expression levels detected was relatively modest, mostly in the 0.5–1.5-fold range. This contrasts with the approximately 50-fold inhibition of *GLI1* and other HH pathway target genes seen in MB treated with SMO inhibitors [41]. The SMO inhibitor concentrations used by Hahn and co-workers (up to 30 µM) were in great excess of the levels required to completely block SMO activity (0.1 µM) [41,97]. These results indicate that SMO-dependent HH pathway activity is absent from RMS tumour cell lines. While the high concentrations of SMO inhibitors used did affect cell growth, the effects observed were variable across the cell lines, they could be positive or negative, and they did not correlate with the effect on *GLI1* expression. As concluded by Hahn and co-workers [97], the observed effects may represent off-target effects of the compounds. This is the most likely explanation for observations of different responses among a class of inhibitors that share the same mechanism of action—inhibition of SMO. As discussed previously, cyclopamine has a narrow therapeutic index, so it can be challenging to distinguish on-target from off-target effects. By contrast, the other SMO inhibitors only exhibited inhibitory effects on tumour cell growth when used at concentrations several hundredfold higher than those required to achieve target inhibition. These very high doses are unlikely to be achieved in patients, and, if they could be achieved, they may well be accompanied by off-target toxicities. Thus, these studies do not support the use of SMO inhibitors for the treatment of RMS.

The fact that heterozygous loss of *Ptch1* in mice is associated with ERMS, depending on the genetic background, demonstrates that HH pathway activation is capable of driving the initiation of RMS, even if it is not prevalent in human cancer [92]. The insensitivity of these tumours to treatment with cyclopamine led to the proposal that, while initiation of ERMS may be HH pathway-dependent, during tumour progression dependency on HH pathway signalling is lost [31]. Interestingly, while expression of an activated *Smo* gene was shown to drive BCC and MB formation in cell lineages that are both HH-expressing and HH-responsive, ERMS arises from cell lineages in which the HH pathway is not active [105]. Taken together, the results indicate that ERMS is in a distinct category from BCC and MB, which clearly harbour an activated HH pathway. Nevertheless, the presence of high levels of GLI1 in ERMS may provide an opportunity for agents such as GANT61, even if SMO inhibitors are not recommended [95]. A similar situation may exist in rhabdoid tumours that express GLI1 but do not harbour HH pathway mutations [62]. These tumours arise as a consequence of loss of SNF5, a chromatin remodelling component, which can directly bind to GLI1 [62]. Similar to ERMS, cyclopamine was not able to inhibit tumour growth, whereas ATO did show some activity [106].

## 10. Resistance mechanisms

**Figure 3.**
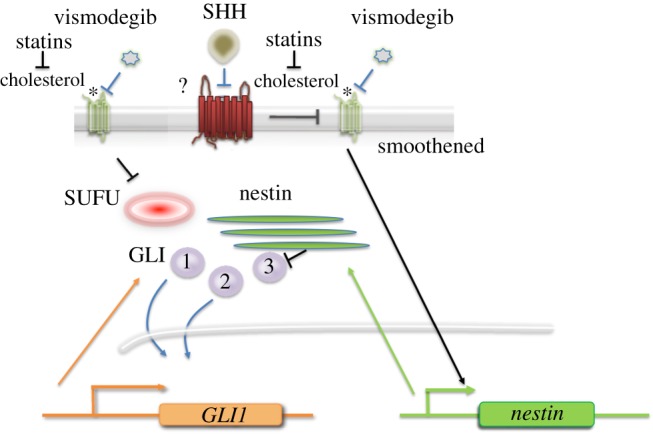
The paradoxical HH pathway. SHH, secreted by tumour-associated astrocytes, induces nestin expression in tumour cells lacking PTCH1, through a signalling mechanism that requires SMO. Nestin accumulates in tumour cells and binds to GLI3, thereby abrogating a negative feedback loop, leading to increased HH pathway activity. Both the induction of GLI1 expression in the absence of PTCH1 and SHH-induced nestin expression require the presence of cholesterol. Statins and vismodegib synergize in the inhibition of HH pathway activity.

The Achilles heel of precision therapeutics is the ease with which drug resistance can develop. This was evident in the first clinical trial of SMO inhibitors, in which a patient with advanced metastatic MB relapsed after an initial dramatic response to treatment [73]. Molecular analysis of a recurrent tumour biopsy revealed the presence of a point mutation in *SMO* (D473H) that reduced the affinity for vismodegib. Interestingly, the same mutation was observed in a study designed to model drug resistance in mouse allograft tumours [73]. The finding that the amino acid substitution effectively blocked drug binding essentially proved the mechanism of action of vismodegib and it was a harbinger of things to come (figure 3). In BCC, drug resistance arises primarily a consequence of activating mutations in *SMO* [72,107], whereas no *SMO* mutations were detected in three cases of HH-MB that had acquired resistance to vismodegib [50]. Mutations in the HH pathway that lie downstream of SMO, including loss of SUFU and amplification of GLI1/2, also confer resistance to SMO inhibitors [72,107]. This was also predicted from GEM model studies [25,71]. Thus, acquired resistance to SMO inhibitors in BCC and MB develops, at least in part, through alterations that bypass the role of SMO in driving HH pathway activity (figure 3).

An additional hypothesis, first proposed by investigators from Novartis Inc., suggested that upregulation of IGF-1/PI3 K signalling compensates for loss of HH pathway activity in MB that acquire resistance to SMO inhibitors [108]. However, the authors reported that while PI3 K inhibitors appeared to prevent the development of resistance to the SMO inhibitor LDE-225, they were not able to inhibit the growth of established tumours. In addition, while loss of PTEN in *Ptch1^+/−^* mice results in MB that respond to SMO inhibitors by stopping growth, the treated tumours fail to regress [109]. The PI3 K pathway has long been proposed to contribute to MB growth [110], PTEN mutations have been reported in human MB [111] and heterozygous ablation of PTEN in mice carrying a *SmoA1* transgene promotes MB formation [112]. Recently, the effect of PTEN loss on MB formation was shown to occur in cells within the postnatal perivascular progenitor niche [113]. Taken together, these results support a role for the PI3 K pathway in MB, but it remains unclear as to whether or not this represents a mechanism for driving resistance to SMO inhibitors. Nevertheless, the observations point to the need to consider additional signalling pathways as potential targets for co-treatment of HH pathway MB and BCC with SMO inhibitors. In both cases, numerous additional genetic lesions are present in the tumours and these may represent potential drivers that could be targeted using precision therapeutics [60,114].

The persistence of the HH pathway gene expression signature in resistant tumours indicates that, at least in part, the resistance mechanism involves pathway activation at, or downstream of, SMO. Recently, Oro and co-workers [115] investigated the mechanism whereby GLI1 transcription activity is increased in mouse allograft BCCs selected for resistance to SMO inhibitors. In resistant tumours lacking activating mutations in SMO, they found that GLI1 participates in a complex with SRF and MKL1 which promotes enhanced transcription of HH pathway target genes. They also show that MKL1 accumulates in the nucleus as a consequence of cytoskeletal activation of RHO. The findings suggest that MKL1 inhibitors may be effective in treating a subset of HH pathway tumours that are resistant to SMO inhibitors and they imply that the combined use of SMO inhibitors and MKL1 inhibitors should be considered for the treatment of naive tumours [115]. These tumours would also be predicted to be sensitive to agents that target GLI proteins similar to GANT61 [74].

## 11. Cholesterol and the Hedgehog pathway

Cholesterol plays several roles in the HH pathway. It is a necessary modification of HH ligands [116] that is required for long-range signalling [117]. Initially, it was speculated that abnormal cholesterol metabolism could affect SHH function, thereby leading to holoprosencephaly [118]. However, the recent identification of cholesterol as the endogenous ligand for SMO [119,120] provides a compelling alternative mechanism. The first clue regarding a direct effect of sterols on SMO was the observation that certain cholesterol derivatives, oxysterols, could stimulate HH pathway activity [121]. However, it was not clear that the abundance and affinity of these compounds was sufficient to allow binding *in vivo*. Structural studies confirmed the interaction of cholesterol itself with the extracellular domain of SMO [122]. These findings immediately raised the question of whether the clinically approved inhibitors of cholesterol metabolism, statins, would be effective in treating HH pathway tumours.

Attempts had already been made to investigate the potential of statins alone, or in combination with the SMO inhibitor cyclopamine, for the treatment of MB and other tumours thought to be dependent on HH pathway activity [89,123–126]. These studies, for the most part, used tumour cell lines which, as discussed above, fail to maintain an active HH pathway [25]. The effects observed required the use of high concentrations of statins, around 1000-fold higher than those required to block cholesterol biosynthesis. Similarly, cyclopamine was used at a concentration that causes off-target toxicity [28]. The limitations of these experimental approaches mean that the data did not provide proof-of-concept support for the use of statins in the treatment of HH pathway tumours.

**Figure 4.**
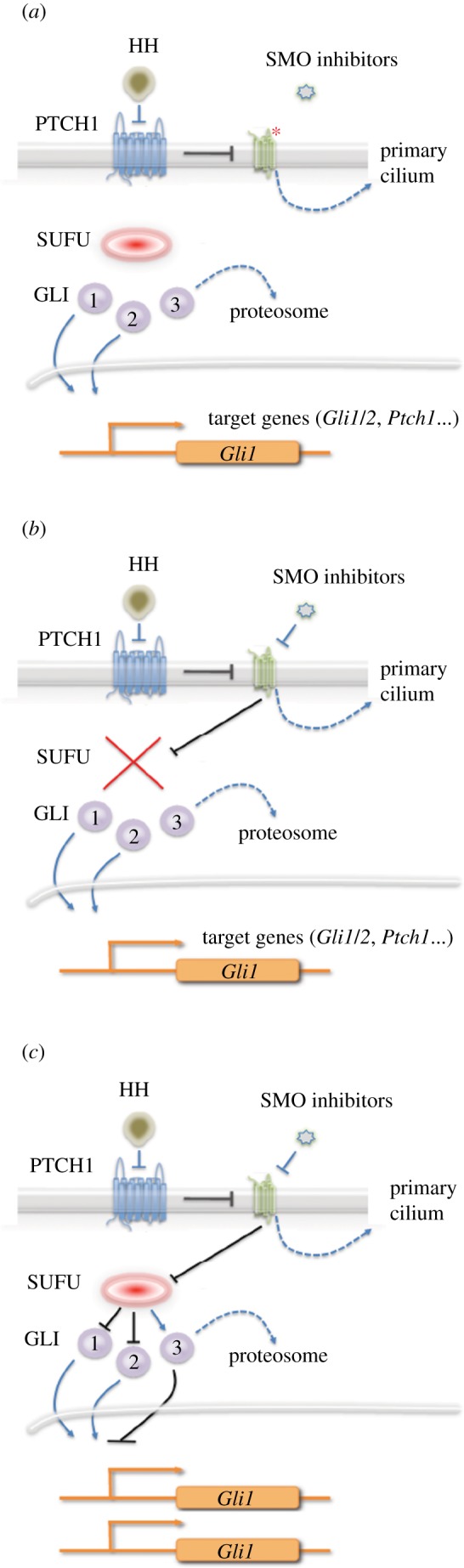
Mechanisms of resistance to SMO inhibitors. Three mechanisms of acquired resistance to SMO inhibitors have been identified in tumours, all of which result in downstream activation of the pathway. (*a*) Mutation of SMO in the drug-binding site leads to loss of inhibition. (*b*) Loss of SUFU leads to downstream activation of the pathway. (*c*) Amplification of *GLI1*, or *GLI2*, leads to increased expression and downstream activation of the HH pathway.

Recently, Yang and co-workers [127] demonstrated that statins do indeed function synergistically with SMO inhibitors in the treatment of MB. They observed that cholesterol biosynthesis is upregulated in HH-MB from both mice and humans. The inhibition of cholesterol biosynthesis, using physiological levels of simvastatin, atorvastatin or triparanol, all reduced HH pathway activity and proliferation of tumour cells. Simvastatin or atorvastatin alone reduced the growth of allograft mouse MB and they functioned synergistically, together with low doses of vismodegib, to prevent tumour growth [127]. These findings support the use of statins in the treatment of HH pathway tumours in conjunction with SMO inhibitors, and they suggest that some resistant tumours may still be sensitive to statin treatment. Currently, SMO inhibitors are not recommended for use in young children because of their effects on bone growth [10,11,13]. Potentially, the combined use of statins may allow the use of lower doses of SMO inhibitors to avoid bone toxicity while still being effective as antitumour agents (figure 4).

## 12. Conclusion

BCC and MB cancers that harbour an activated HH pathway should be considered as a distinct class from other tumours in which the HH pathway has been proposed as a therapeutic target. Even in BCC and MB, the decision to include a SMO inhibitor among the therapeutic options depends on the exact nature of the HH pathway activating mutation and also, potentially, the epigenetic mechanisms responsible for maintaining pathway activity. The most significant take-home message from this review is that there is a lack of compelling preclinical evidence supporting the use of SMO inhibitors in a broad class of tumours lacking mutations in HH pathway genes. Several factors, including limitations in the model systems used, the experimental designs and overinterpretation of marginal results, conspired to present unrealistic expectations regarding the potential impact of SMO inhibitors on human cancer. It remains possible that agents that bind GLI1/2 directly could be used in the tumours that express high levels of these proteins regardless of the presence of activating mutations in the HH pathway. In addition, GLI1/2 inhibitors may also be effective in tumours harbouring activating mutations in SMO, or downstream mutations in the HH pathway, as well as in tumours that have acquired resistance to SMO inhibitors.

Data obtained using human tumour cell lines treated with SMO inhibitors were not predictive in the clinic because the HH pathway was suppressed in cell culture. In the case of mouse MB, the absence of the tumour microenvironment, specifically astrocytes that secrete *SHH*, was not present in the cell culture conditions employed. Potentially, this could be addressed by defining appropriate co-culture systems to preserve the tumour microenvironment and HH pathway activity *in vitro* [25,26]. Xenograft models, including patient-derived xenograft models, also failed to provide reliable preclinical data. While allografts of mouse MB closely resemble the original spontaneous tumours, as they re-create a supportive tumour microenvironment in the flank of immunosuppressed mice, this has not yet been successful in the case of human xenografts. Therefore, in HH BCC and MB, xenograft models are not recommended for preclinical studies.

**Figure 5.**
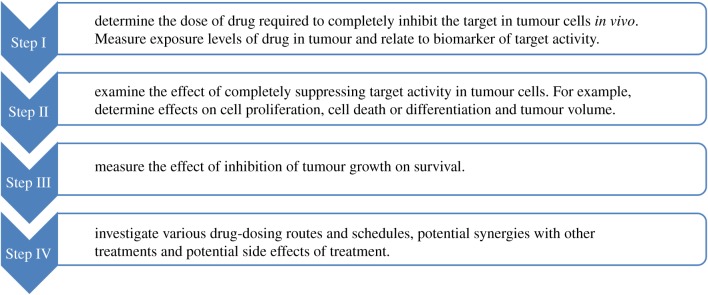
The use of GEM models. The use of GEM models for the development of SMO inhibitors followed a multi-step path that serves as a guideline for future drug development. Step I involves determining the level of inhibitor required to block the target in spontaneous tumours *in vivo*. This required the use of quantitative biomarkers as a read-out of pathway activity. Step II measures the consequences of target inhibition in terms of tumour growth. Step III assesses whether inhibition of tumour growth results in increased lifespan. Step IV represents follow-up studies to refine dosing regimens, identify drug-resistance mechanisms, explore potential co-treatments and investigate side effects.

**Figure 6.**
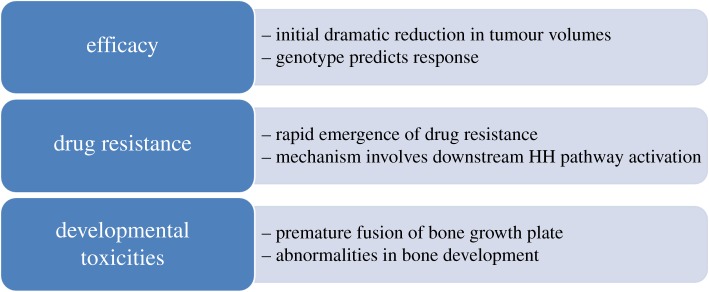
SMO inhibitor predictions from mouse GEM models. A number of observations were made in GEM models that paralleled the experience with SMO inhibitors in the clinic. There was an initial dramatic elimination of large tumour mass. The tumour genotype accurately predicted the response to treatment. There was a rapid acquisition of resistance due to downstream activating mutations in the HH pathway. Developmental toxicities were observed in bone growth in young mice and young children.

The use of GEM mice, particularly mice with a loss-of-function *Ptch1* mutation, was critical for the development of SMO inhibitors. The strategy developed for the successful use of this class of models for the development of SMO inhibitors can be applied more broadly (figure 5). Briefly, the first step is to determine if the drug can bind and inhibit the target *in vivo*. This initial step should use a physiological route of delivery (e.g. oral gavage) and the experiments should be conducted quantitatively to determine the dose regime that maintains target suppression. To be successful, this initial *in vivo* study requires the use of robust biomarkers to monitor pathway activity in tumour tissues. The second step is to determine the effect of target suppression on tumour growth. This requires a combination of molecular, pharmacological and histopathological methodology. While understanding the mechanism of action of the agent under consideration is not required, it is extremely useful to support future development. In the case of SMO inhibitors, the primary mechanism appeared to be inhibition of tumour cell proliferation, leading to abortive differentiation and ultimately cell death [41]. By contrast, the off-target effects of SMO inhibitors used at high, non-physiological concentrations resulted in direct induction of apoptosis [28]. The third step is to determine whether the effects of the agent on tumour growth result in increased survival. This step requires prolonged dosing which should be carried out using a physiological route that models the intended delivery system anticipated in clinical studies. The models can also be used for follow-up studies that optimize dosing regimens, identify resistance mechanisms, explore drug combinations and analyse potential side effects of prolonged treatment. Using these approaches, a series of predictions were made based on the GEM models that were recapitulated in clinical trials of SMO inhibitors (figure 6).

**Figure 7.**
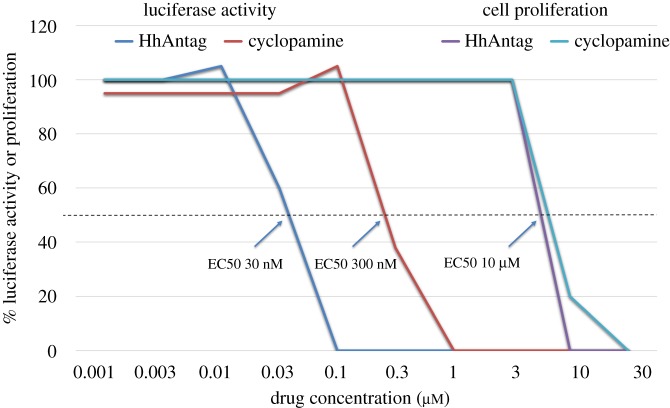
Dose–response analysis. This illustration is based on data reported in [41]. Using a GLI1-luciferase assay in NIH3T3 cells, the dose of HhAntag (a SMO inhibitor) and cyclopamine, required to inhibit 50% of SHH-induced HH pathway activity, was determined to be 30 and 300 nM, respectively. By contrast, the dose of each agent required to block proliferation of tumour cell lines by 50% was approximately 10 µM. These data, by themselves, demonstrate that there is no relationship between the effect of the agents on HH pathway activity and tumour cell proliferation because the doses are so discrepant. Although simple, this key lesson was widely ignored because of unconscious bias.

Many of the issues involving the lack of reproducibility of preclinical research, carried out using SMO inhibitors, appear to stem from the fact that several inhibitors were used at high concentrations where they caused toxic off-target effects, resulting in false-positive data. This was particularly challenging in the case of the naturally occurring SMO inhibitor, cyclopamine, because it exhibits a very narrow therapeutic index. In the case of some of the new, highly potent, SMO inhibitors, these toxic effects were only seen when the drugs were used in vast excess (several hundredfold) over the levels required to suppress HH pathway activity. Figure 7 illustrates the relative differences of specific and non-specific dose–response curves. This is a very important, fundamental principle in pharmacology. When the dose of a drug reaches a level that saturates the target, adding more drug does not increase the specific response. This is a particular concern in cancer research as the assays used for determining drug responses involve inhibition of cell proliferation or induction of cell death, both of which can readily arise from off-target, non-specific effects. In addition, traditionally, classic chemotherapeutic agents were developed to be used at the maximum tolerated dose. This concept was developed for broadly active chemotherapeutics, for example, those that induce DNA damage, to cause as much damage as possible to cancer cells while preserving life. However, this strategy is not appropriate for targeted therapies which should be used at the dose specifically required to block the target in the tumour.

The route of drug administration is also key. It is rarely, if ever, valid to test the toxic potential of anticancer drugs by direct inoculation of small allografts or xenografts. In this case, the concentration of drug at the site of inoculation is extremely high and it is not really possible to conduct a dose–response analysis. In addition, because small allografts and xenografts are not yet established as transplanted tumours, the assay really only addresses the ability of the agent tested to inhibit the establishment of a graft, rather than its ability to cause tumour regression. The biological processes involved in the establishment of a tumour graft may well be very different from those responsible for maintaining tumour growth; therefore, the results obtained are not relevant to the treatment of human cancer.

Despite the approval of SMO inhibitors for the treatment of BCC in the USA, the National Institute for Health and Care Excellence (NICE) in the UK did not recommend the use of vismodegib for symptomatic metastatic, or locally advanced, BCC. This decision was based on overall survival data and an estimate that the cost-effectiveness of vismodegib, compared with best supportive care, is much higher than 30 000 UK pounds per quality-adjusted life-year gained. This analysis did not include the use of molecular diagnostics to select only those tumours for treatment that would be predicted to respond. The committee that made the recommendation acknowledged that the overall survival data were immature, that previous trials were not applicable to the UK population and that, while clinically relevant benefits are plausible, they did not find the evidence presented as substantial. The main issue appears to be the insistence on the use of overall survival data as the primary criteria for recommendation without due consideration of impacts on health-related quality of life. At the same time, it was acknowledged by the committee that mortality is rarely attributed to locally advanced BCC in the average UK population. There remains some hope, as data accumulate from sites outside of the UK supporting the benefits of SMO inhibitors in specific patient populations, that this decision will be revisited. It will also be important to include companion diagnostics to rule out patients that would not be predicted to benefit from SMO inhibitors either initially or after the appearance of drug-resistant mutations.

## 13. Future directions

The translational journey of SMO inhibitors teaches us that there is no ideal, single approach or perfect model. Human cancer is diverse, and it is not possible to represent all of that diversity in collections of cell lines, transplanted tumours or GEM models. However, specific models can be used very effectively to address defined proof-of-concept questions. Cell lines with reporter constructs allowed precise determination of the drug concentrations required to fully suppress HH pathway activity. Spontaneous tumours in GEM models allowed analysis of the ability of compounds to penetrate the blood–brain barrier, inhibit the HH pathway in tumour cells and to show antitumour efficacy only in the appropriate molecular subtype of tumour. Allograft models permitted analyses of the mechanisms involved in tumour elimination, and they predicted mechanisms of drug resistance that were recapitulated in the clinic. These same models are now being used to investigate drug combinations aimed at different targets in the HH pathway or complementary targets that could be used together with SMO inhibitors. In the case of BCC, there is great interest in developing topical applications and mice may not provide a suitable model because mouse skin is more permeable than human skin [128–130]. Recent advances in the ability to determine tumour genetic profiles from circulating tumour DNA suggest that the molecular subtype of paediatric MB can be determined from blood biospecimens, opening the possibility that SMO inhibitors could be used even prior to surgery, radiation or chemotherapy, to avoid the side effects of these traditional approaches [131]. As these studies progress, it is important that investigators maintain constant vigilance against unconscious bias. We also need to investigate our failures in the context of tumour biology to understand their root causes. As we all know, if we fail to learn lessons from history, we may well be doomed to repeat it.

## Data accessibility

This article does not contain any additional data.
